# Supplementary material for: Intelligent wearable allows out-of-the-lab tracking of developing motor abilities in infants
Source: Commun Med (Lond). 2022 Jun 15;2:69. doi: 10.1038/s43856-022-00131-6 (PMC9200857; doi:10.1038/s43856-022-00131-6)
Supplement: Supplementary file 1 — Description of Additional Supplementary Files [file 43856_2022_131_MOESM1_ESM.pdf]

Description of Additional Supplementary Files

File Name: Supplementary Data 1

Description: The raw figure data has been made available in Supplementary Data 1
