# Supplementary material for: Intelligent wearable allows out-of-the-lab tracking of developing motor abilities in infants
Source: Commun Med (Lond). 2022 Jun 15;2:69. doi: 10.1038/s43856-022-00131-6 (PMC9200857; doi:10.1038/s43856-022-00131-6)
Supplement: Supplementary file 2 — Supplementary Materials [file 43856_2022_131_MOESM2_ESM.pdf]

Supplementary material

List of supplementary figures:

Supplementary Figure S1: Compounded inter-rater agreement confusion matrices.
Supplementary Figure S2: Inter-rater agreement and classifier performance for posture-conditional movement categories.
Supplementary Figure S3: Decision tree diagram of the active carrying detection (ACD) annotations.
Supplementary Figure S4: Specificity and recall confusion matrices for the ACD classifier.
Supplementary Figure S5: Block diagram depicting the procedure to obtain MAIJU recording distributions.
Supplementary Figure S6: Compounded confusion matrices for all annotation versus classifier output pairs.
Supplementary Figure S7: Compounded confusion matrices for all classifier training targets (IAR processed labels) versus classifier output pairs.
Supplementary Figure S8: Comparison of algorithmic motor ability quantitation to human annotations.
Supplementary Figure S9: Bland-Altman plots for the algorithmic annotations vs. classification error.
Supplementary Figure S10: Training and usage of the BABA Infant Motor Score (BIMS) classifier from MAIJU distributions.
Supplementary Figure S11: Correlation between motor ability track probabilities vs. age at recording.
Supplementary Figure S12: Correlation between motor ability track probabilities vs. Alberta Infant Motor Scale (AIMS).
Supplementary Figure S13: Correlation between true and predicted AIMS scores.
Supplementary Figure. S14: Transition rates between posture and movement categories and their relationship to infant age.

List of supplementary tables:

Supplementary Table S1. Annotation guidelines for track A: Posture

Supplementary Table S2. Annotation guidelines for track B: Movement

Supplementary Table S3. Annotation guidelines for track C: Time periods to be excluded from classifier training

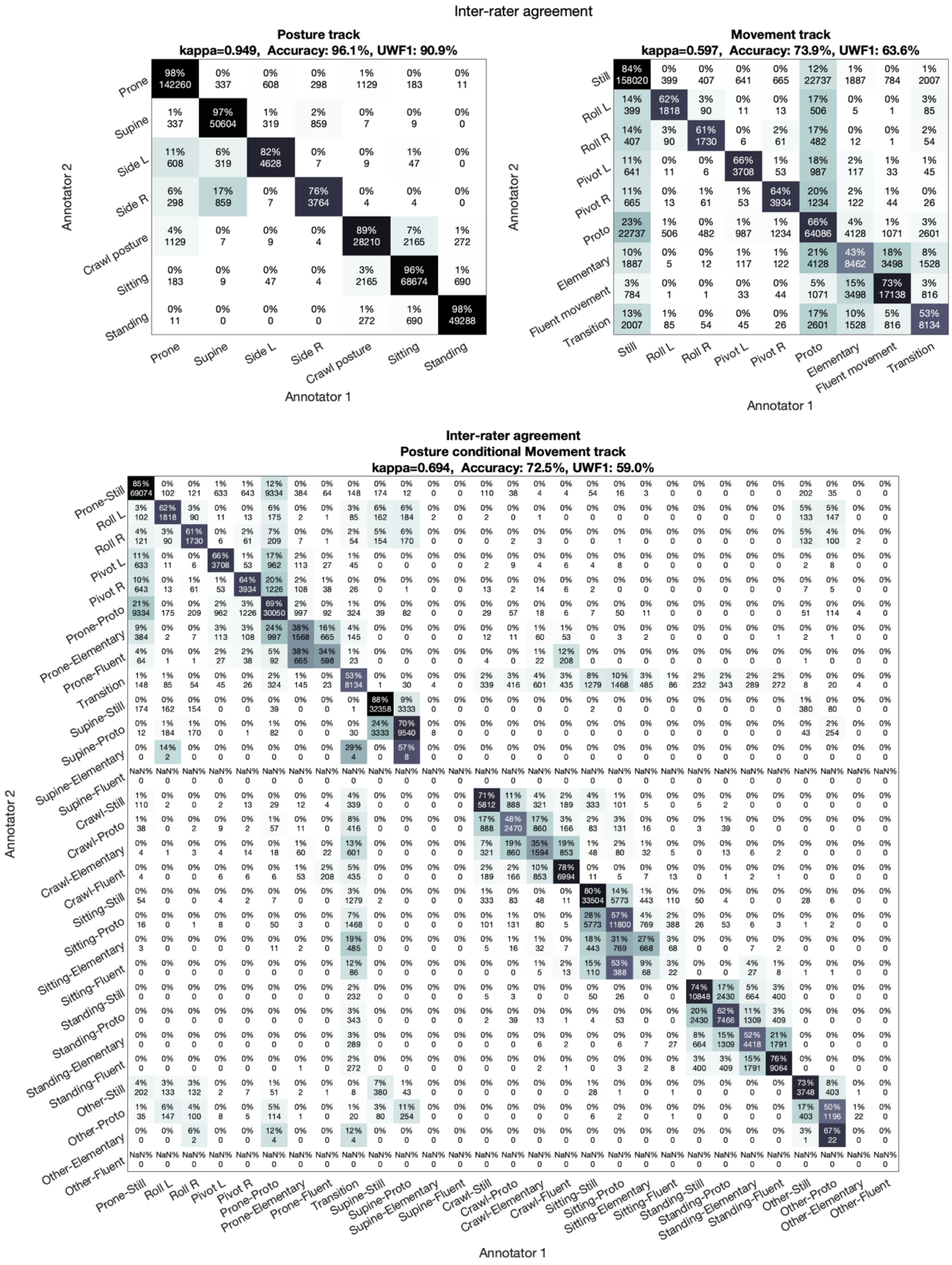

Supplementary Figure S1: Compounded inter-rater agreement confusion matrices *for posture (top left), movement (top right), and posture-conditional movement tracks (bottom). The percentages and colors in cell (i,j) give the recall probability of category i being annotated by Annotator 2 given annotation to cateogry j by Annotator 1. The plain values show the raw frame counts in the confusion matrix.*

		Still			Proto			Elementary			Fluent		
**Standing**	Proportion (%)	4,8			4,4			2,7			3,4		
	Kappa	0,73	0,71	0,83	0,61	0,62	0,75	0,51	0,5	0,65	0,75	0,76	0,84
	F1 (%)	74	72	83	62	64	76	52	52	66	76	76	85
	UWF1 (%)	87	86	91	80	81	88	75	75	82	88	88	92
	Accuracy (%)	98	97	98	97	97	98	98	97	98	98	98	99
**Sitting**	Proportion (%)	13,6			4,5								
	Kappa	0,78	0,77	0,87	0,54	0,52	0,68						
	F1 (%)	80	80	89	58	57	70						
	UWF1 (%)	89	89	94	77	76	84						
	Accuracy (%)	95	95	97	95	95	97						
**Crawl**	Proportion (%)	2,8			1,5			2,1			2,9		
	Kappa	0,7	0,63	0,72	0,47	0,39	0,47	0,34	0,4	0,6	0,78	0,78	0,87
	F1 (%)	71	64	73	47	40	48	35	45	61	78	79	87
	UWF1 (%)	85	82	86	73	69	73	67	72	80	89	89	94
	Accuracy (%)	99	98	98	98	98	98	98	98	98	99	99	99
**Prone**	Proportion (%)	21,4			8			1,8			0,2		
	Kappa	0,81	0,82	0,9	0,64	0,57	0,71	0,37	0,3	0,45	0,34	0,18	0,25
	F1 (%)	85	85	92	69	61	73	38	35	46	34	18	26
	UWF1 (%)	90	91	95	82	79	86	69	67	73	67	59	63
	Accuracy (%)	93	94	97	92	93	96	99	98	99	99,4	99,5	99,7
**Supine**	Proportion (%)	9,6			2,8								
	Kappa	0,87	0,87	0,94	0,69	0,65	0,79						
	F1 (%)	88	88	94	70	66	80						
	UWF1 (%)	93	94	97	84	83	90						
	Accuracy (%)	98	98	99	98	98	99						

	Transition			Pivot			Roll		
Proportion (%)	5,7			4,2			2		
Kappa	0,51	0,51	0,68	0,64	0,61	0,71	0,62	0,6	0,73
F1 (%)	53	54	70	65	61	72	61	62	74
UWF1 (%)	76	79	84	82	80	86	81	81	87
Accuracy (%)	96	95	96	99	99	99	99	99	99

Human vs Human
Raw annotations vs Classifier
Processed annotations vs Classifier

Supplementary Figure S2: Inter-rater agreement and classifier performance for posture-conditional movement categories. Reported number represent the proportion of the data within data annotated dataset (Proportion %), Fleiss' kappa (Kappa), F1-score (F1), unweighted average F1 (UWF1 %), and accuracy (Accuracy %). The performance metrics are reported for raw human vs human annotations (red), raw human annotations vs classifier output (blue), and IAR-processed human annotations vs classifier output (black).

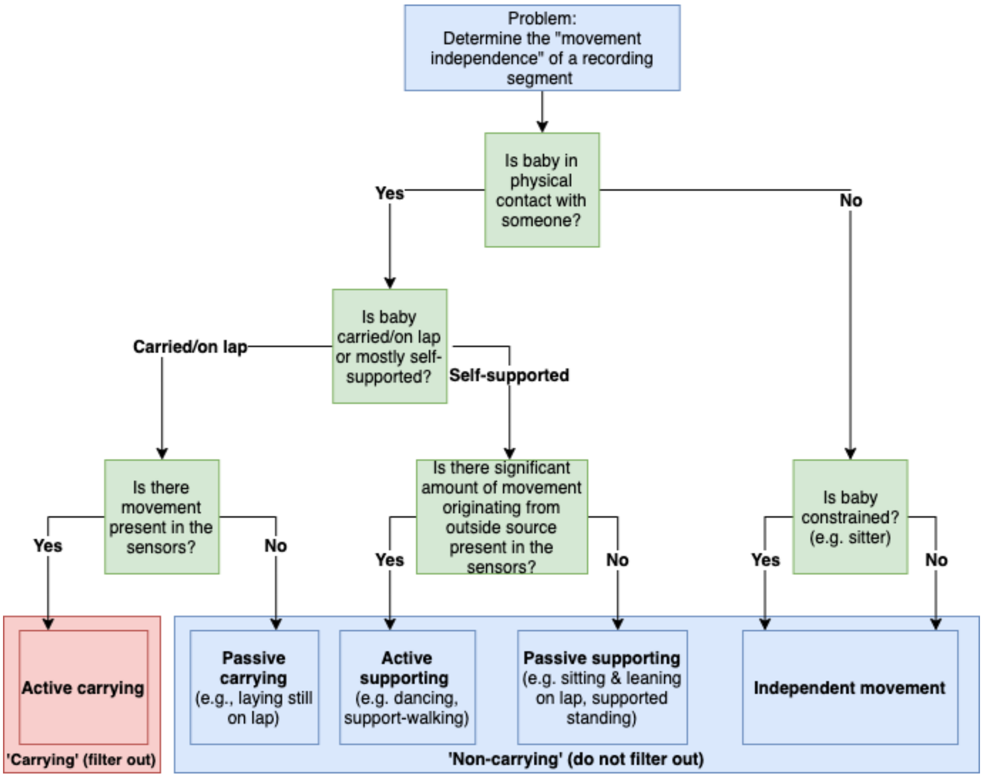

Supplementary Figure S3: Decision tree diagram of the active carrying detection (ACD) annotations. The annotations were produced into five separate categories, which were fused into binary categories. Additional category boundary combinations were explored, but the ACD was deemed as the most robust in terms of performance.

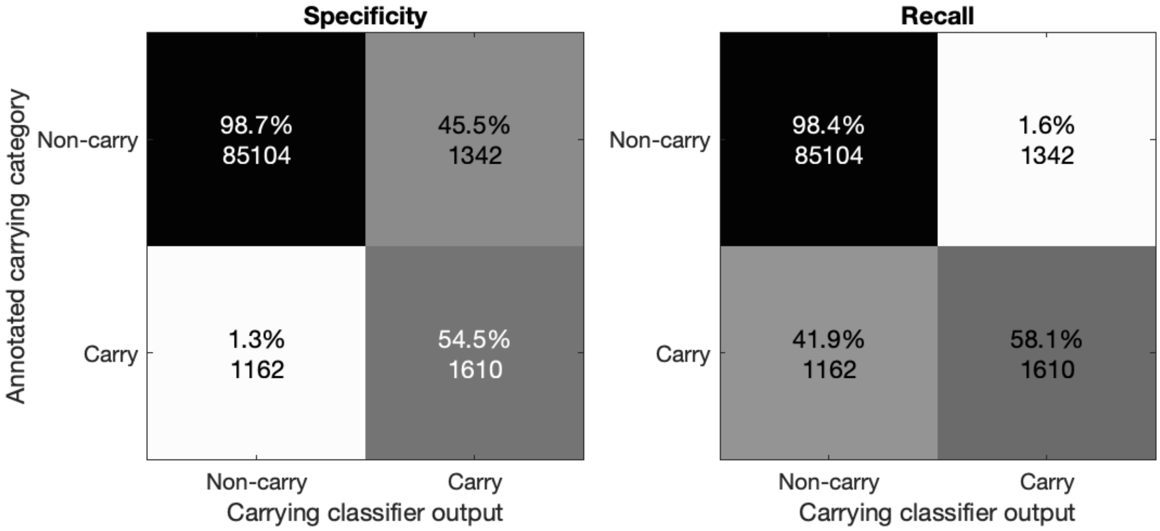

Supplementary Figure S4: Specificity and recall confusion matrices for the ACD classifier.

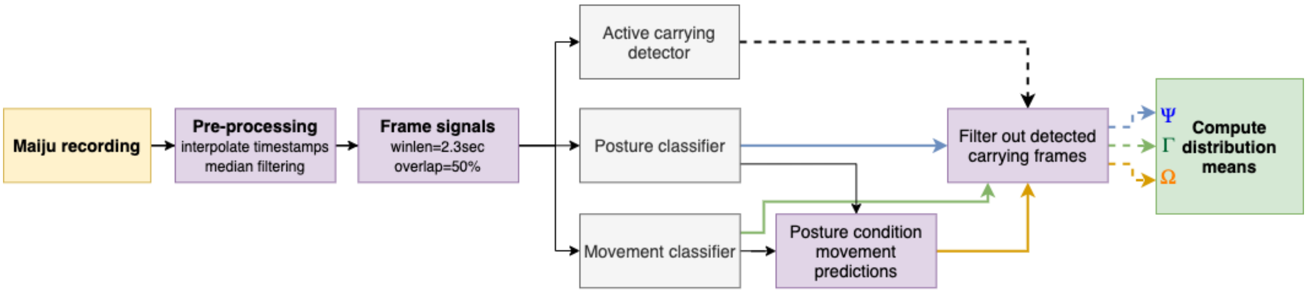

Supplementary Figure S5: Block diagram depicting the procedure to obtain MAIJU recording distributions for downstream analysis. Psi=posture distribution used in Figs. 2C, 3C, 4A-E; Gamma=Movement distribution used in Figs. 2C, 3C; Omega=posture-conditioned movement distribution used in Figs. 4A-D. The classifiers produce a one-hot coded vector (1, N_{cats}) for each frame, where N_{cats} is the number of output categories in the classifier. Thus, for each recording, the classifiers output a matrix of shape (N_{frames}, N_{cats}), where N_{frames} is the number of 2.3sec (120 sample) frames in the recording. The ACD classifier produces a binary mask of shape (N_{frames}, 1), which is applied as a filter to the posture and movement classifier outputs before computing the distributions. The distribution for each track is computed by taking the mean of the ACD-filtered one-hot matrices along the frame axis to yield a vector of shape (1, N_{cats}).

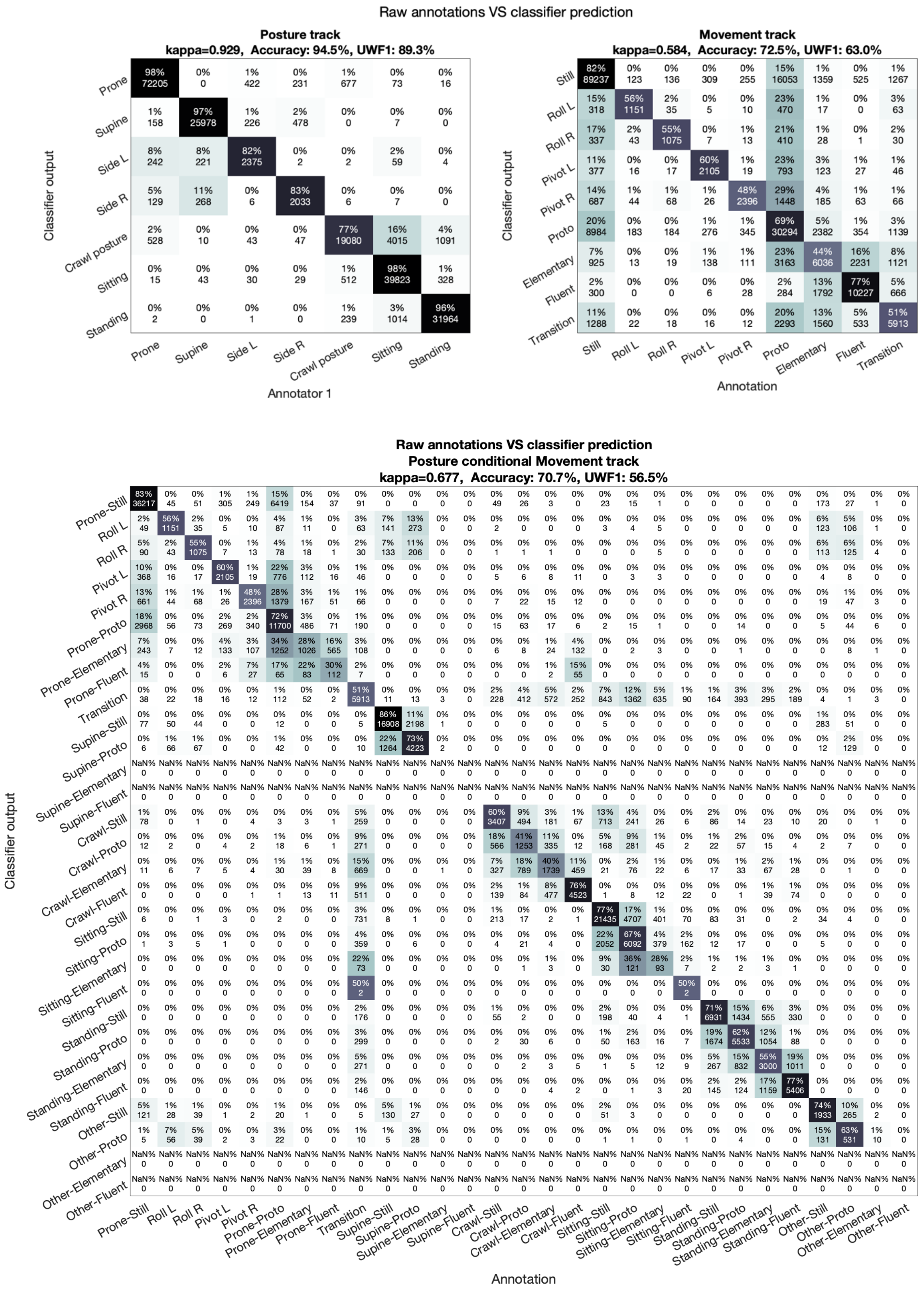

Supplementary Figure S6: Compounded confusion matrices for all annotation versus classifier output pairs. Top left: Posture categories; Top right: Movement categories; Bottom: Posture-conditioned movement categories. The percentages (and colorings) in cells (i,j) give the recall probability of category i being classified by the classifier given annotation to cateogry j by an annotator. The plain values show the raw frame counts in the confusion matrices.

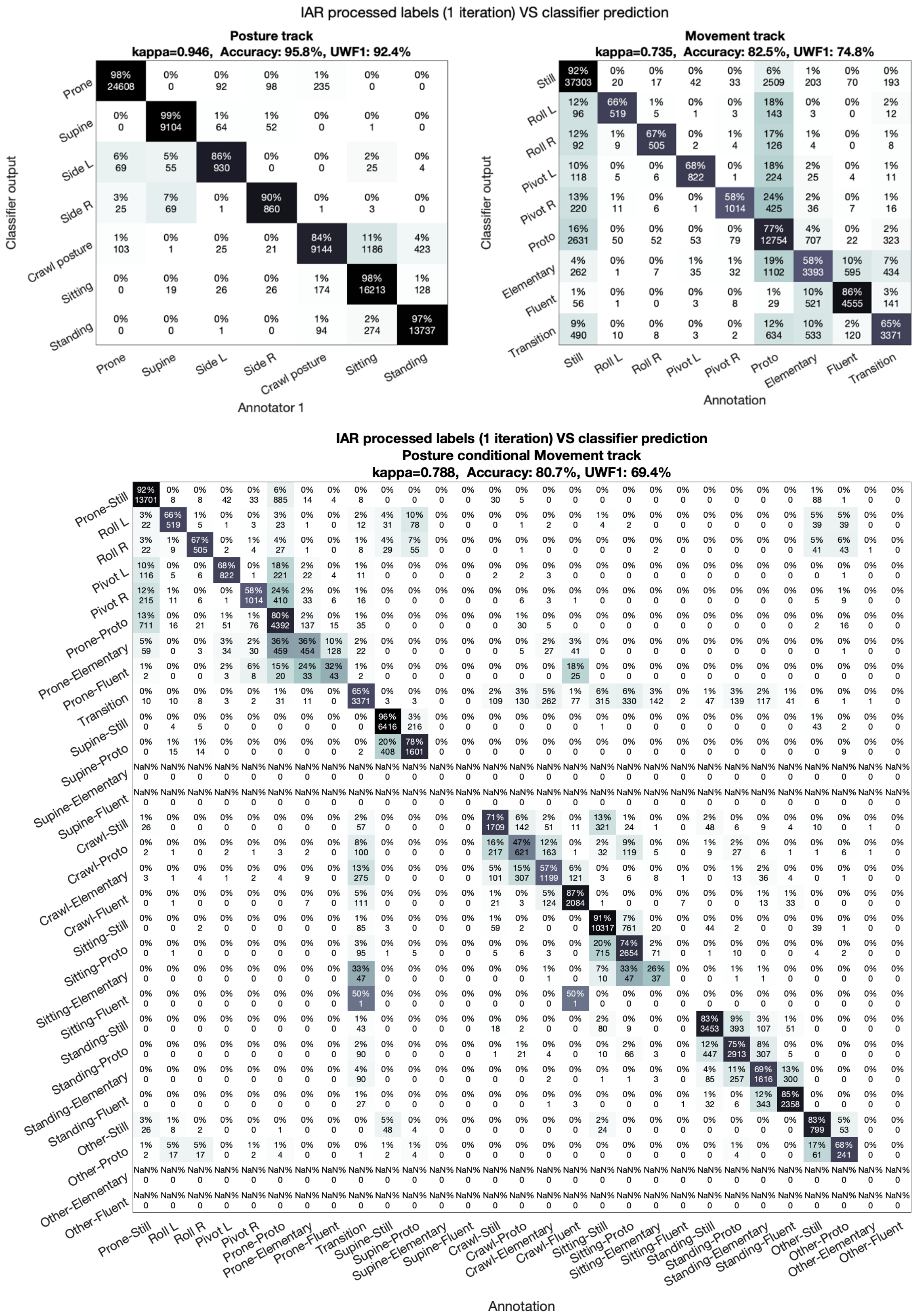

Supplementary Figure S7: Compounded confusion matrices for all classifier training targets (IAR processed labels) versus classifier output pairs. Top left: Posture categories; Top right: Movement categories; Bottom: Posture-conditioned movment categories. The percentages (and colorings) in cells (i,j) give the recall probability of category i being classified by the classifier given target to category j in the training targets. The plain values show the raw frame counts in the confusion matrices.

(A)

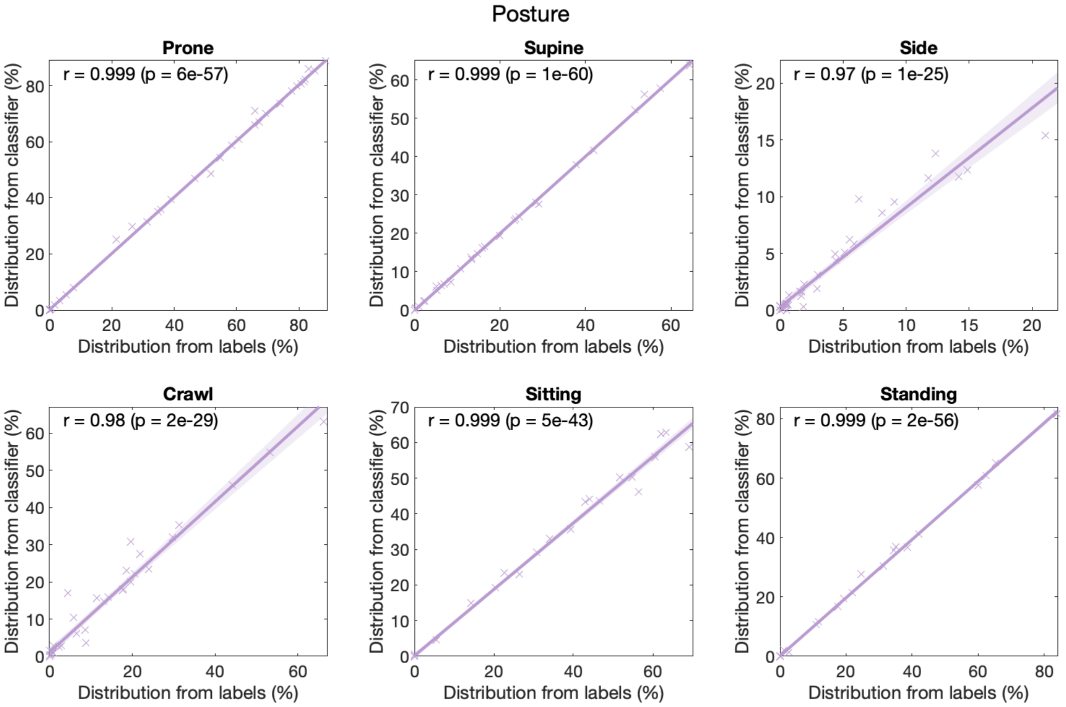

(B)

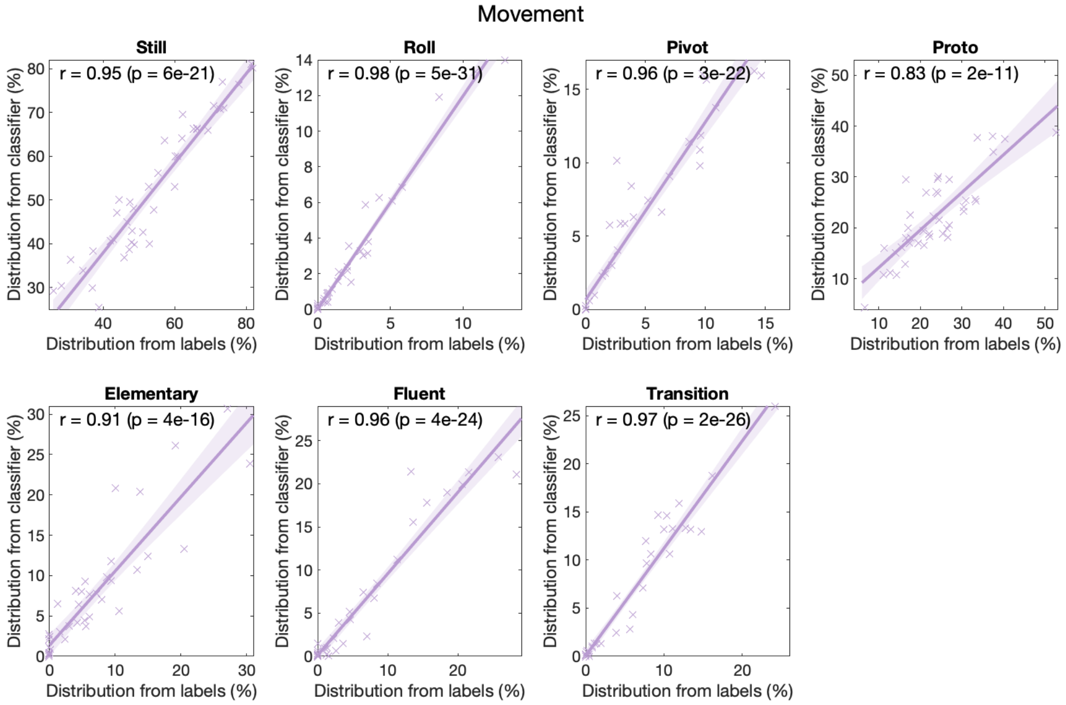

Supplementary Figure S8: Comparison of algorithmic motor ability quantitation to human annotations. The scatter plots show the proportion of time spent in the given postures (A) or movements (B) in individual infants. The values are estimated by the classifier algorithm (Y axis) and compared to the human visual annotations (X axis). The Pearson's r (and its p-value) denotes the linear correlation between the proportion values.

(A)

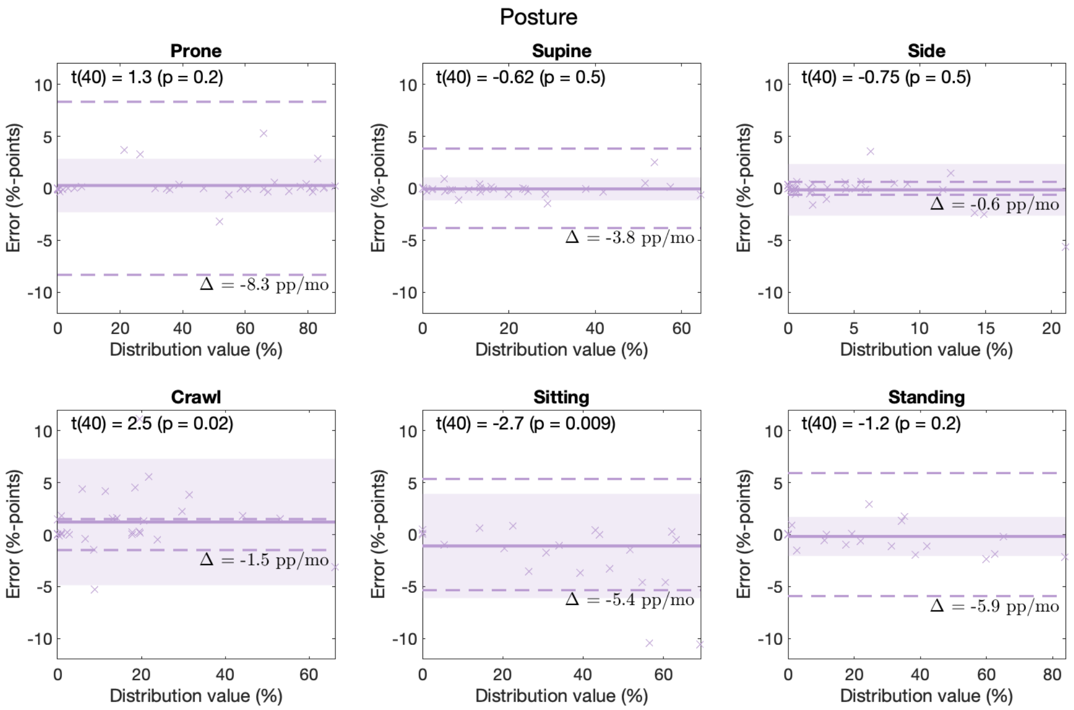

(B)

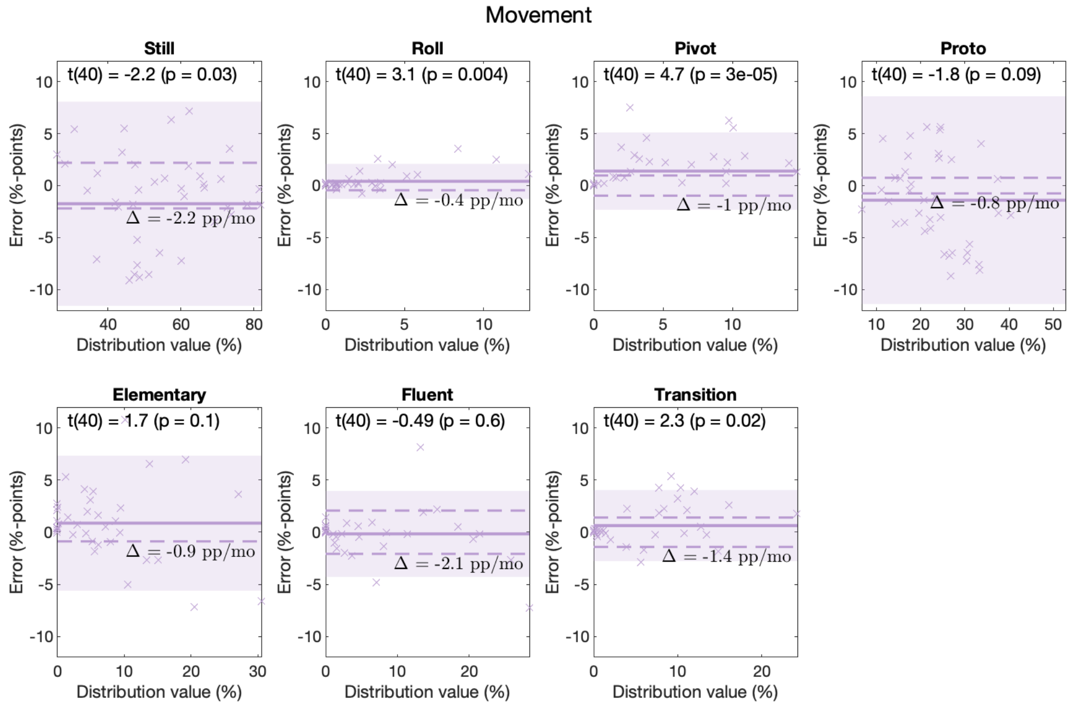

Supplementary Figure S9: Bland-Altman plots for the algorithmic annotations vs. classification error (A) for posture and (B) for movement categories. The stippled lines depict the rate of change (percentage points per month) of a linear regression model fitted between the age (in months) and given motor ability occurrence (cf. Fig. 2C). Note that the stippled lines contain 100% of the measurements in the posture categories, and 88% of the measurements for fluent movement. The shaded zone depicts the 95% confidence interval (in percentage points) of the classifier error. The t-value depicts the two-tailed t-test result on the null hypothesis that the error has a mean of zero; this shows that the proportional estimates are unbiased.

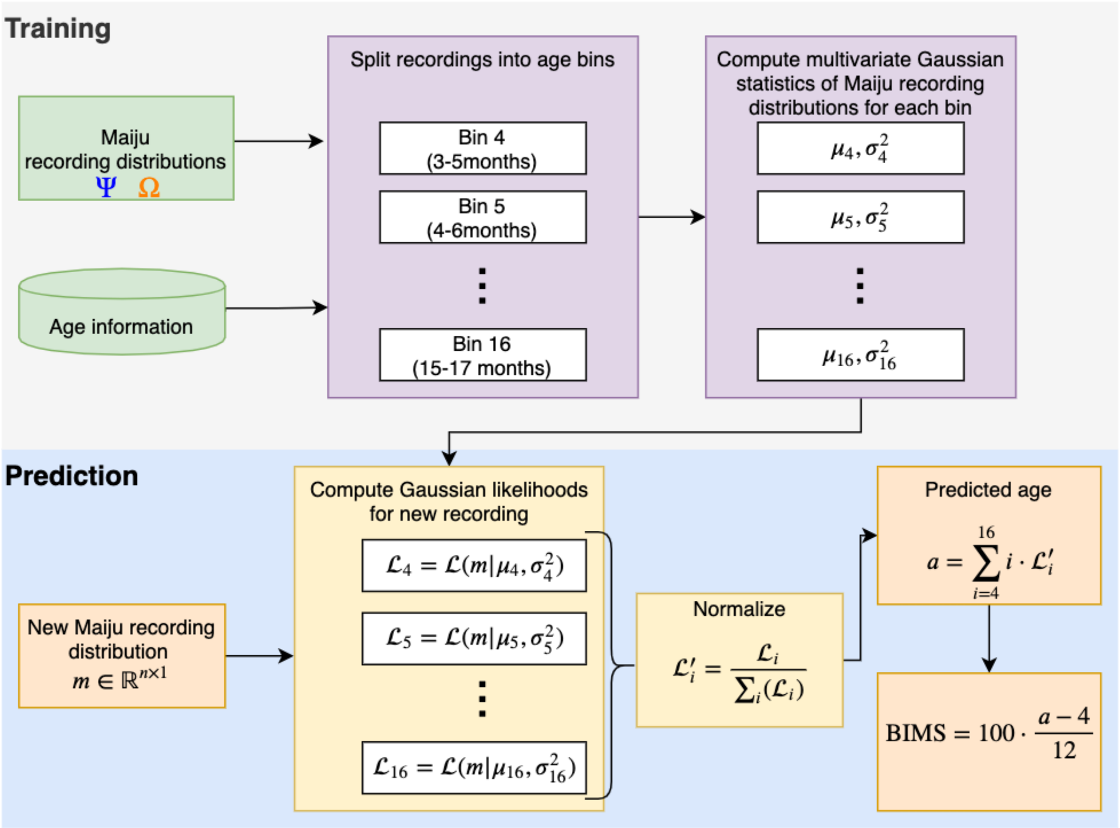

Supplementary Figure S10: Training and usage of the BABA Infant Motor Score (BIMS) classifier from MAIJU distributions. Each age bin was set to have a minimum number of three recordings. Missing recordings were filled with closest recordings (by age). Each variance component was set to have a minimum value of 10^{-4}.

(A)

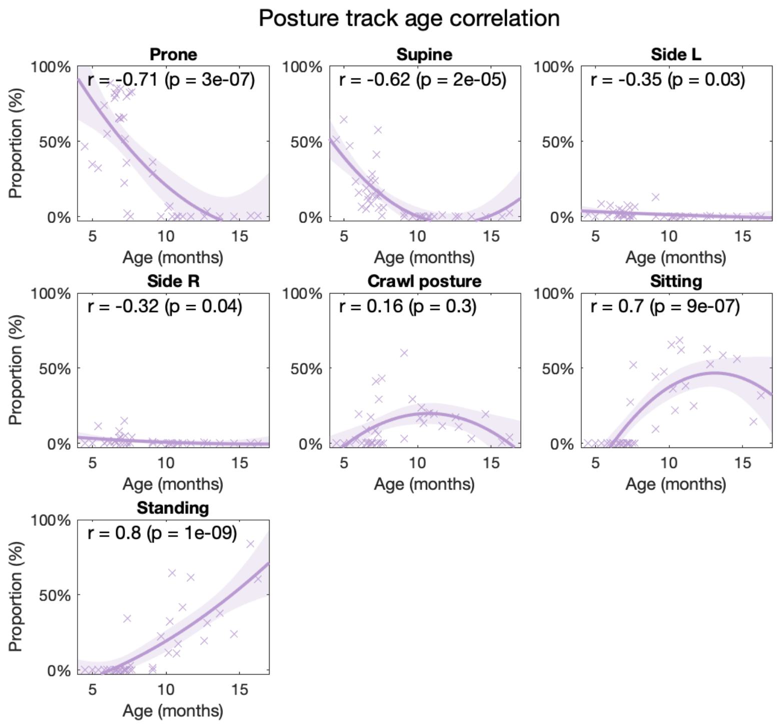

(B)

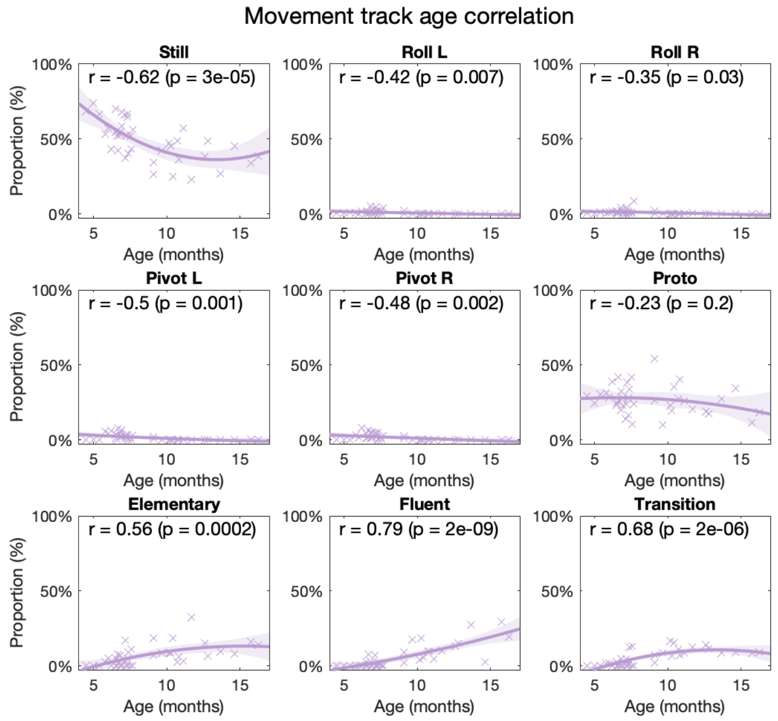

Supplementary Figure S11: Correlation between motor ability track probabilities vs. age at recording for each A) posture and B) movement category as identified from the video recordings by the human observers (individual points) alongside a quadratic regression model (solid line) with its 95% confidence interval (colored area). Pearson's r and its p-value denotes the linear correlation of the data.

(A)

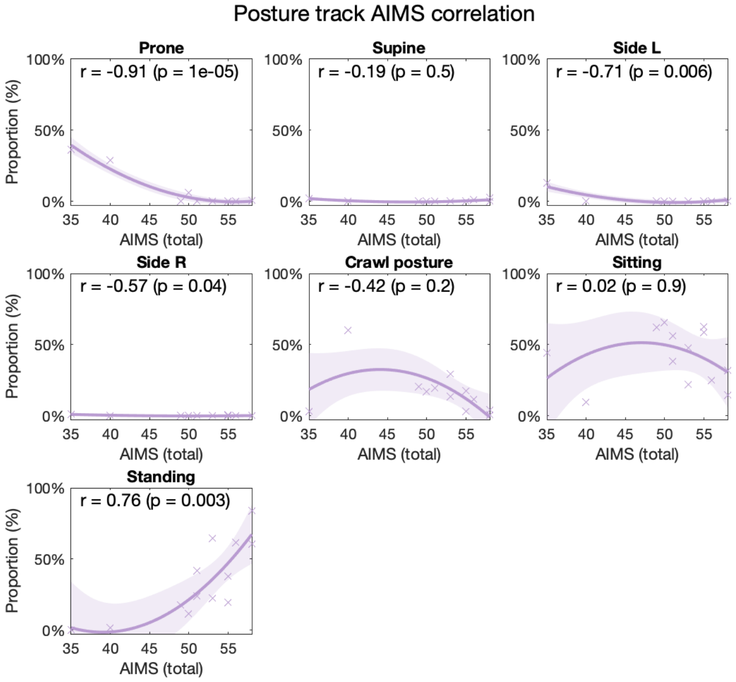

(B)

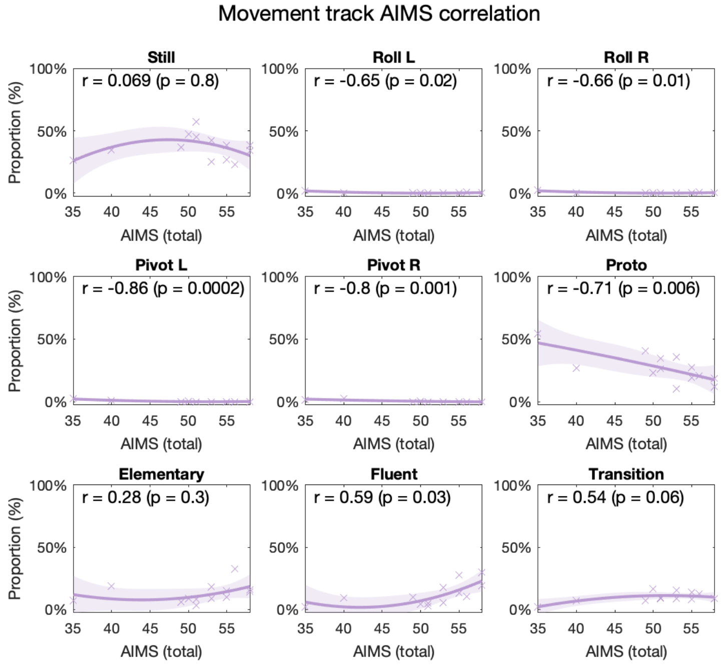

Supplementary Figure S12: Correlation between motor ability track probabilities vs. Alberta Infant Motor Scale (AIMS) for each A) posture and B) movement category as identified from the video recordings by the human observers (individual points) alongside a quadratic regression model (solid line) with its 95% confidence interval (colored area). Pearson's r and its p-value denotes the linear correlation of the data.

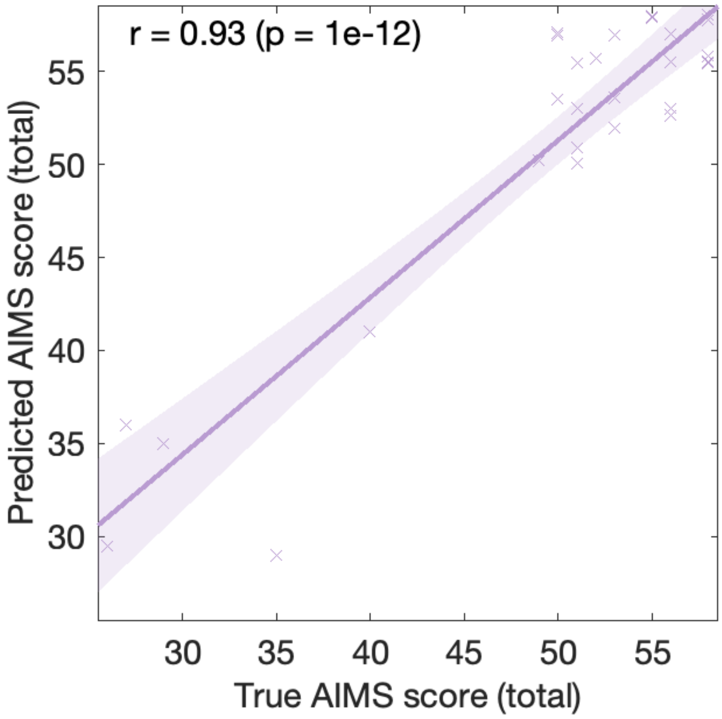

Supplementary Figure S13: Correlation between true and predicted AIMS scores. The AIMS score classifier was trained similarly to the BIMS classifier, where the age groups were replaced by AIMS score groups. The predictions were obtained with LOSO cross-validation.

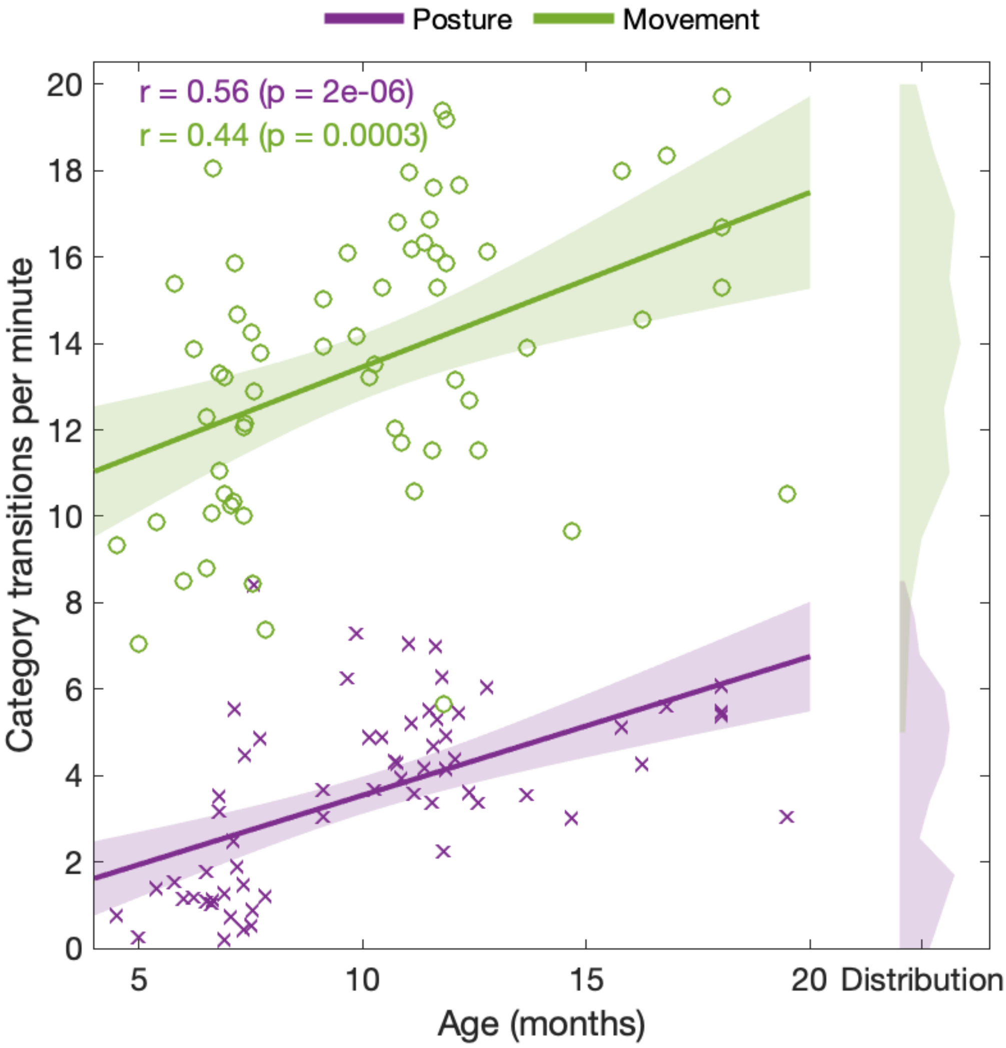

Supplementary Figure. S14: Transition rates between posture and movement categories and their relationship to infant age. A high rate of transitions was observed within both the posture (purple; avg 3.6/min, IQR 1.5-5.2) and movement categories (green; avg 13.5/min, IQR 10.7-16.1). Notably, both transition rates also showed a statistically significant positive correlation to infant age, indicating developmentally increasing temporal variability in motor ability.

Supplementary Table S1. Annotation guidelines for Track A: Posture. The percentage values are the (F1 score) probabilities of the given category (agreement between Annotators 1 and 2). Main source of confusion between postures comes from the transitory movements (i.e. where the posture changes to another posture), especially when the infant spends longer periods in such posture between clear-cut categories.

Category name	Definition	Inter-rater reliability & common confusions	Borderline cases of the category
Prone	Lying belly-side down, without significant support from the limbs.	In general, a very distinct category (98%). The most common confusions are crawl (~1%) and side (~1%) postures.	• Extended arm support (upper trunk lifted with hands, hips on the ground)
Supine	Lying on the back (belly-side up).	In general, a very distinct category (97%). The most common confusions are side postures (~3%).	–
Side left/right	Lying on the left/right side of the trunk, with the trunk on the ground. Left/right orientation is determined by the infant's point of view, which direction is down.	The most ambiguous posture category (79%). Most common confusions are between prone (~9%) and supine (~12%), primarily caused by the inherently continuous nature of the categories within the vertical axis of rotation.	–
Crawl	A posture with a horizontally aligned trunk that is off the ground, actively supported by the arms and legs.	A fairly distinct posture category (89%). The most common confusions are prone (~4%) and sitting (~7%) postures as infants commonly transit from crawling to these.	• Bear walking posture (whole trunk lifted & supported with hands and feet)<br>• Forward lean, typically demonstrated from knee sitting (horizontal trunk, support on hands)
Sitting	A stance with a vertically aligned trunk, sustained by bottom on the ground (without active support from the hands).	In general, a very distinct category (96%). The most common confusions are crawl (~3%) and standing (~1%) postures: Infants might lean on their hands while sitting or standing where their trunk is more horizontally aligned, i.e. the borderline between the categories is inherently continuous.	• Side lean (horizontal trunk, bottom on the ground, support on one side's arm)<br>• Knee sitting with bottom on the ground
Standing	A stance with a vertically aligned trunk, supported by the legs (feet or knees), with hands off the ground. Includes both supported and unsupported conditions.	In general, a very distinct category (98%). The most common confusions are crawl (~1%) and sitting (~1%) postures: From standing, infants often move to crawling or sitting and vice versa.	• Knee standing with bottom lifted from the ground<br>• Supported standing (e.g., with a trolley, onto furniture)

Supplementary Table S2. Annotation guidelines for Track B: Movement. The percentage values are the (F1 score) probabilities of the given category (agreement between Annotators 1 and 2). Main sources of confusion in movement annotations are the less clear-cut category boundaries, which in the motility spectrum resembles the task of "naming colors in a rainbow".

Applicable posture context	Category name	Description	Inter-rater reliability & common confusions	Borderline cases of the category
All postures	Still	The infant is not actively moving. Small amounts of isolated limb movements are allowed.	The most distinct movement category (84%). Most commonly confused with proto movement (~12%), which is conceptually the closest movement category.	• Softly playing with a toy in hand
	Proto	Active movement that does not change the infant's position or posture.	Average-level inter-rater agreement (66%). Most commonly confused with still (~23%), elementary (~4%), and transition (~3%).	• Shifting of weight bearing within posture (e.g., sitting angle)<br>• Repeated rudimentary or preparatory movement iterations or patterns, typically symmetrical across limbs. E.g., infant jiggles in a newly acquired posture<br>• Active playing with a toy
	Elementary	Inefficient movement that changes the infant's position.	The most ambiguous movement category (43%) as it contains emerging movement patterns easily confused with proto movement (21%) and stillness (10%) on one end of the motility spectrum, and fluent movement (18%) on the other.	• Distinct movement patterns of limbs (e.g. steps) that do not result in positional movement<br>• Shaky yet continuous movement (e.g., unstable balancing during walking)<br>• Pivoting-like movement that also results in forward movement
	Fluent	Efficient movement that changes the infant's position. Movement that has multiple recurring cycles of fluent-appearing, rhythmic movement patterns.	A fairly distinct movement category (73%). The most common confusions are elementary movement (~15%), proto movement (~5%), transition (~3%), and still (~3%).	–
	Transition	Movement that changes the infant's posture where movement cycles are not recurring (excluding rolling), e.g., sitting ↔ standing, crawling ↔ standing, etc.	A fairly ambiguous movement category (53%) containing transitory movement across all of the main posture categories. Thus easily confused with macro still (The most ambiguous movement category (~13%), proto (~17%), elementary (~10%), and fluent (~5%) movement.	• Falling or tumbling (e.g., from standing)
Supine/ prone/ side postures	Roll left/right	Rotation along the vertical axis that changes the infant's posture along the prone, side, and supine categories. Left/right direction of rotation is determined from the infant's point of view.	Average-level inter-rater agreement (62%). Rolling is typically observed in young infants (ages between 4 and 8 months) and therefore the most common confusions are proto movement (~17%), and still (~14%).	• Partial rolling (prone ↔ side, side ↔ supine)
Prone posture	Pivot left/right	Rotation along the sagittal axis that changes facing direction without a change in position per se. Left/right direction of rotation is determined from the infant's point of view.	Average-level inter-rater agreement (65%). Pivoting is typically observed in young infants (ages between 4 and 8 months) and therefore the most common confusions are proto movement (~19%), and still (~11%).	• "Proto"-pivoting (clear rotational attempts along the sagittal axis)<br>• Pivoting can also result in the change of posture (e.g., prone ↔ side)

Table S3. Annotation guidelines for Track C: Time periods to be excluded from classifier training.

Category name	Description
Exclude	Events that are not taken into further analysis (e.g., recording suit is taken off the baby or the baby is being carried)
Out of screen	The infant is out of view in the video recording, and the annotation cannot be made.
