# Supplementary material for: Intelligent wearable allows out-of-the-lab tracking of developing motor abilities in infants
Source: Commun Med (Lond). 2022 Jun 15;2:69. doi: 10.1038/s43856-022-00131-6 (PMC9200857; doi:10.1038/s43856-022-00131-6)
Supplement: Supplementary file 5 — Reporting Summary [file 43856_2022_131_MOESM5_ESM.pdf]

nature portfolio

Corresponding author(s): Manu Airaksinen
Sampsa Vanhatalo

Last updated by author(s): May 13, 2022

Reporting Summary

Nature Portfolio wishes to improve the reproducibility of the work that we publish. This form provides structure for consistency and transparency in reporting. For further information on Nature Portfolio policies, see our Editorial Policies and the Editorial Policy Checklist.

Statistics

For all statistical analyses, confirm that the following items are present in the figure legend, table legend, main text, or Methods section.

n/a	Confirmed	
☐	☒	The exact sample size (n) for each experimental group/condition, given as a discrete number and unit of measurement
☐	☒	A statement on whether measurements were taken from distinct samples or whether the same sample was measured repeatedly
☐	☒	The statistical test(s) used AND whether they are one- or two-sided<br>*Only common tests should be described solely by name; describe more complex techniques in the Methods section.*
☐	☒	A description of all covariates tested
☐	☒	A description of any assumptions or corrections, such as tests of normality and adjustment for multiple comparisons
☐	☒	A full description of the statistical parameters including central tendency (e.g. means) or other basic estimates (e.g. regression coefficient) AND variation (e.g. standard deviation) or associated estimates of uncertainty (e.g. confidence intervals)
☐	☒	For null hypothesis testing, the test statistic (e.g. F, t, r) with confidence intervals, effect sizes, degrees of freedom and P value noted<br>*Give P values as exact values whenever suitable.*
☒	☐	For Bayesian analysis, information on the choice of priors and Markov chain Monte Carlo settings
☒	☐	For hierarchical and complex designs, identification of the appropriate level for tests and full reporting of outcomes
☐	☒	Estimates of effect sizes (e.g. Cohen's d, Pearson's r), indicating how they were calculated

Our web collection on statistics for biologists contains articles on many of the points above.

Software and code

Policy information about availability of computer code

Data collection	The movement data were collected with the wearable device described in the present paper, designed and custom-made in our laboratory. It uses Movesense IMU sensors (Suunto Ltd, Vantaa, Finland). The data was transmitted wirelessly using a mobile application (Kaasa Solution GmbH, Dusseldorf, Germany). In addition, a synchronized video recording was performed to a subset of infants. Neurological assessment of the infants was performed by standard clinical examinations and Alberta Infant Motor Score (AIMS). Additional survey questions were requested from the parents concerning infant's movement behaviour.
Data analysis	Data pre-processing and analysis were performed using custom Matlab codes (version 2021a). The analysis codes are made publicly available at Zenodo (doi:10.5281/zenodo.6417486)<br><br>The video recordings were annotated with Anvil software (version 6.0; https://www.anvil-software.org/).<br><br>The motility classifier was implemented as custom code using Python (version 3.6.9) and Tensorflow (version 1.12.0). The motility classifier can be run through BABA cloud (www.babacloud.fi) with credentials that are freely available at request (M.A.).

For manuscripts utilizing custom algorithms or software that are central to the research but not yet described in published literature, software must be made available to editors and reviewers. We strongly encourage code deposition in a community repository (e.g. GitHub). See the Nature Portfolio guidelines for submitting code & software for further information.

Data

Policy information about availability of data

All manuscripts must include a data availability statement. This statement should provide the following information, where applicable:

- Accession codes, unique identifiers, or web links for publicly available datasets
- A description of any restrictions on data availability
- For clinical datasets or third party data, please ensure that the statement adheres to our policy

The data underlying figures 1b, 2b-c, 4b-c, and 5a-e can be found from Supplementary Data 1. An example data-set of three recordings is made publicly available at doi:10.5281/zenodo.6417486 25. The other original (raw) movement data can be made available upon request to the authors (S.V.). The use of this dataset in further scientific work will require a data sharing agreement with Helsinki University Hospital. Processed data, such as motility classifier outputs, can be made available upon request.

Field-specific reporting

Please select the one below that is the best fit for your research. If you are not sure, read the appropriate sections before making your selection.

☒ Life sciences ☐ Behavioural & social sciences ☐ Ecological, evolutionary & environmental sciences

For a reference copy of the document with all sections, see nature.com/documents/nr-reporting-summary-flat.pdf

Life sciences study design

All studies must disclose on these points even when the disclosure is negative.

Sample size	This was an exploratory methods-developing study with no prior determination of sample size.
Data exclusions	For the development of classifiers, we used data from N=41 infants (total 29 hours) with synchronized video recordings. Out of the wearable recordings (total length 71.5 hours) we used only periods where the infant was moving spontaneously (total 69 hours). Infants with neurodevelopmental deviance (N=4) were omitted from the development of BIMS and all correlative measures of infant development.
Replication	This is a unique clinical data set of infants using a novel wearable system. There are no available replication datasets yet. To ensure that our findings can be reliably reproduced, we used leave one subject out (LOSO) validation in our algorithmic testing, as per current standards in machine learning field. In addition, we used alternative machine learning strategies, such as self-supervised learning, to ensure parts of the findings.
Randomization	This is not a randomized treatment study.
Blinding	Blinding is not relevant to this study because it is not a randomized treatment study.

Reporting for specific materials, systems and methods

We require information from authors about some types of materials, experimental systems and methods used in many studies. Here, indicate whether each material, system or method listed is relevant to your study. If you are not sure if a list item applies to your research, read the appropriate section before selecting a response.

Materials & experimental systems

n/a	Involved in the study	
☒	☐	Antibodies
☒	☐	Eukaryotic cell lines
☒	☐	Palaeontology and archaeology
☒	☐	Animals and other organisms
☐	☒	Human research participants
☐	☒	Clinical data
☒	☐	Dual use research of concern

Methods

n/a	Involved in the study	
☒	☐	ChIP-seq
☒	☐	Flow cytometry
☒	☐	MRI-based neuroimaging

Human research participants

Policy information about studies involving human research participants

Population characteristics	This was an exploratory study that used prospective recordings with no prior determination of sample size. The movement were available from a group N=59 infants recorded at 4.5-19.5 months of age. 11 of them were born preterm. Some raw data

	within these cohorts have been previously published (Airaksinen et al 2020), however the scope of this previous work was independent to the current study.
Recruitment	All infants were recruited on a voluntary basis via direct contact, via media advertizing, or through participation in other ongoing studies on neurodevelopment.
Ethics oversight	The study was approved by the Ethics committee and the research permission was granted by New Childrens' Hospital, Helsinki University Hospital, Helsinki, Finland. Informed written consent was received from a guardian before inclusion of an infant into the study.

Note that full information on the approval of the study protocol must also be provided in the manuscript.

Clinical data

Policy information about clinical studies

All manuscripts should comply with the ICMJE guidelines for publication of clinical research and a completed CONSORT checklist must be included with all submissions.

Clinical trial registration	This is a methodological development study and not registered as a clinical trial.
Study protocol	This study on methods development did not have a strict prospective study protocol as in conventional treatment trials. The practical protocol on data collection is described in the manuscript, and the practical lab instructions are available at request (from S.V.)
Data collection	Data was recorded in BABA center, Helsinki Children's Hospital, and at infant's homes in Helsinki, Finland region. Total cohort collection lasted from 12-2018 to 6-2020.
Outcomes	This is not a treatment study and no formative outcome measures were defined. The algorithm performance was benchmarked with infant's known chronological age, the clinical AIMS test, and parental surveys.
